# Malignant Phyllodes Tumor With Left Atrial and Bilateral Pulmonary Metastases Treated Surgically: A Case Report

**DOI:** 10.7759/cureus.100309

**Published:** 2025-12-29

**Authors:** Kanako Naito, Mayu Nakagawa, Yasuto Kondo, Masaomi Fukuzumi, Takafumi Sangai

**Affiliations:** 1 Breast and Thyroid Surgery, Kitasato University Hospital, Sagamihara, JPN; 2 Pathology, Kitasato University Hospital, Sagamihara, JPN; 3 Thoracic Surgery, Kitasato University Hospital, Sagamihara, JPN; 4 Cardiovascular Surgery, Kitasato University Hospital, Sagamihara, JPN

**Keywords:** distant metastasis, intracardiac metastasis, malignant phyllodes tumor, pulmonary metastasis, surgery

## Abstract

We report a rare case of a malignant phyllodes tumor with left atrial and bilateral pulmonary metastases. A woman in her 40s had undergone a tumorectomy for a benign phyllodes tumor of the right breast. Two years later, she developed local recurrence diagnosed as a malignant phyllodes tumor and underwent total mastectomy. At follow-up two years after surgery, computed tomography (CT) revealed bilateral pulmonary metastases with tumor extension into the pulmonary veins and left atrium. Echocardiography showed a mobile left atrial mass. Due to the risk of cardiac complications, surgical resection was performed. The patient underwent left atrial tumor resection, right lower lobectomy, and left upper lobe partial resection. Histopathological findings confirmed metastatic malignant phyllodes tumor in all specimens. Postoperatively, she received five cycles of doxorubicin, but the disease progressed, and she passed away six months after surgery. Intracardiac metastasis from a malignant phyllodes tumor is extremely rare and often fatal. Prompt surgical intervention may be beneficial in select cases. In the future, with the development of effective drug therapy for malignant phyllodes tumors, long-term survival may be expected with multidisciplinary treatment, including surgery, even in cases of distant metastasis.

## Introduction

Phyllodes tumors are rare fibroepithelial breast neoplasms, accounting for only 0.3%-0.9% of all breast tumors [1]. They are histologically classified into benign, borderline, and malignant types [2], with malignant tumors comprising approximately 16%-30% of all phyllodes tumors [3]. Malignant phyllodes tumors are characterized by rapid growth, stromal overgrowth, and a tendency for hematogenous spread, making their clinical behavior difficult to predict. Local recurrence is not uncommon, particularly when surgical margins are inadequate, and repeated recurrence may lead to malignant transformation. Distant metastasis occurs most frequently to the lungs, followed by bone and liver. In contrast, intracardiac involvement is exceedingly rare, with only a small number of cases reported worldwide. Tumor invasion into the pulmonary veins or cardiac chambers can cause life-threatening complications such as obstruction, heart failure, or sudden death, often necessitating urgent intervention. Here, we report a rare case of malignant phyllodes tumor with left atrial and bilateral pulmonary metastases, for which surgical resection was performed. This case highlights the aggressive nature of malignant phyllodes tumors while underscoring the importance of comprehensive imaging evaluation and multidisciplinary surgical decision-making in selected patients.

This case was presented at the 32nd Annual Meeting of the Japanese Breast Cancer Society (Sendai, Japan, July 2024).

## Case presentation

A woman in her 40s with a history of asthma and a family history of breast and gastric cancers initially underwent routine breast screening at a previous hospital in year X. A 2.6 cm mass was detected in the CD region of the right breast and was suspected to be a fibroadenoma. Because the patient declined a biopsy, interval follow-up was continued at the same institution, and no interval changes were observed the following year.

In year X + 3, the patient again presented to the previous hospital after noticing a palpable right breast mass. Core needle biopsy (CNB) performed there confirmed a phyllodes tumor, and a tumorectomy was carried out at that hospital. Histopathology revealed a benign phyllodes tumor.

**Figure 1:**
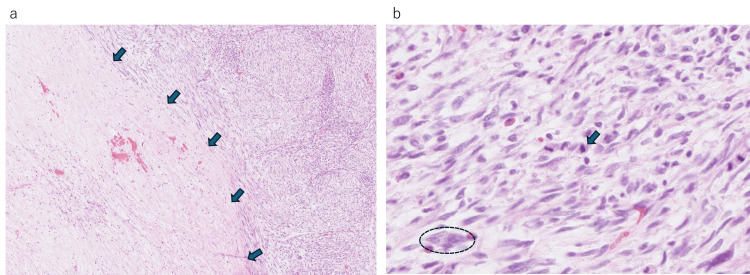
Tumor specimen from the mastectomy at the previous hospital (a) Area inside the arrows shows coagulative necrosis in the tumor center with nuclear loss, while the right side demonstrates variable tumor cell components (H&E, 4x). (b) Densely proliferating spindle cells arranged in an interlacing pattern, showing nuclear atypia (circle) and mitoses (arrow) (H&E, 40x)

Two years later, in year X + 5, she returned to the same hospital with a newly developed right breast mass. Ultrasonography performed at the previous institution suggested recurrence, and repeat CNB showed malignant features. She subsequently underwent a total mastectomy at the previous hospital, and the resected specimen demonstrated a 7.0 × 5.4 × 6.5 cm malignant phyllodes tumor (Figure 1).

In year X + 7, bilateral pulmonary nodules were detected during routine follow-up at the previous hospital. After relocating, the patient was referred to our institution for further evaluation and treatment.

**Figure 2:**
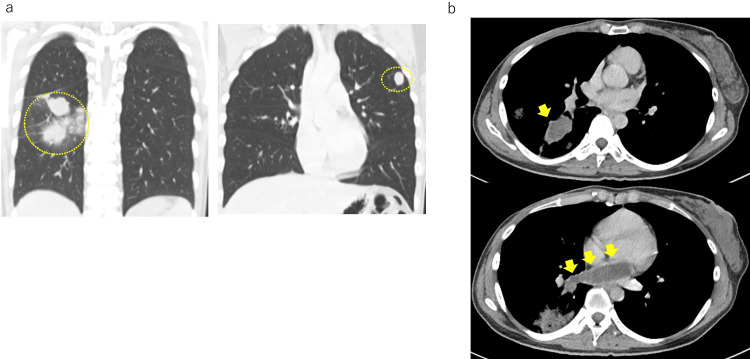
Computed tomography (CT) image of the metastatic lesion (a) Bilateral pulmonary metastases. (b) Tumor invasion into the pulmonary veins and progression within the vasculature

**Figure 3:**
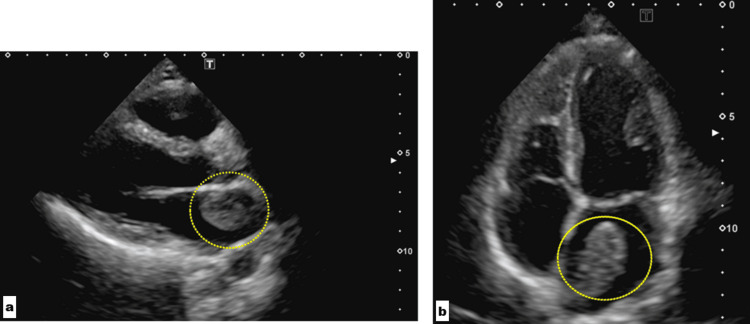
Echocardiographic image of the left atrium nodule (a, b) The mobile mass with heterogeneous internal echoes measuring 43 × 25 mm in the left atrium (inside circles)

Chest computed tomography (CT) at our institution showed bilateral pulmonary metastases with tumor invasion into the pulmonary veins and intravascular extension (Figure 2). Transthoracic echocardiography revealed a mobile, heterogeneous mass measuring 43 × 25 mm within the left atrium (Figure 3). Given the life-threatening risk of intracardiac obstruction, the limited safety and efficacy of chemotherapy in the presence of an intracardiac mass, and the patient’s relatively young age, surgical resection was recommended after multidisciplinary discussion.

A joint cardiothoracic and thoracic surgical team performed left atrial tumor resection, right lower lobectomy, and partial resection of the left upper lobe. The operation lasted six hours and 52 minutes, with a total blood loss of 488 mL.

**Figure 4:**
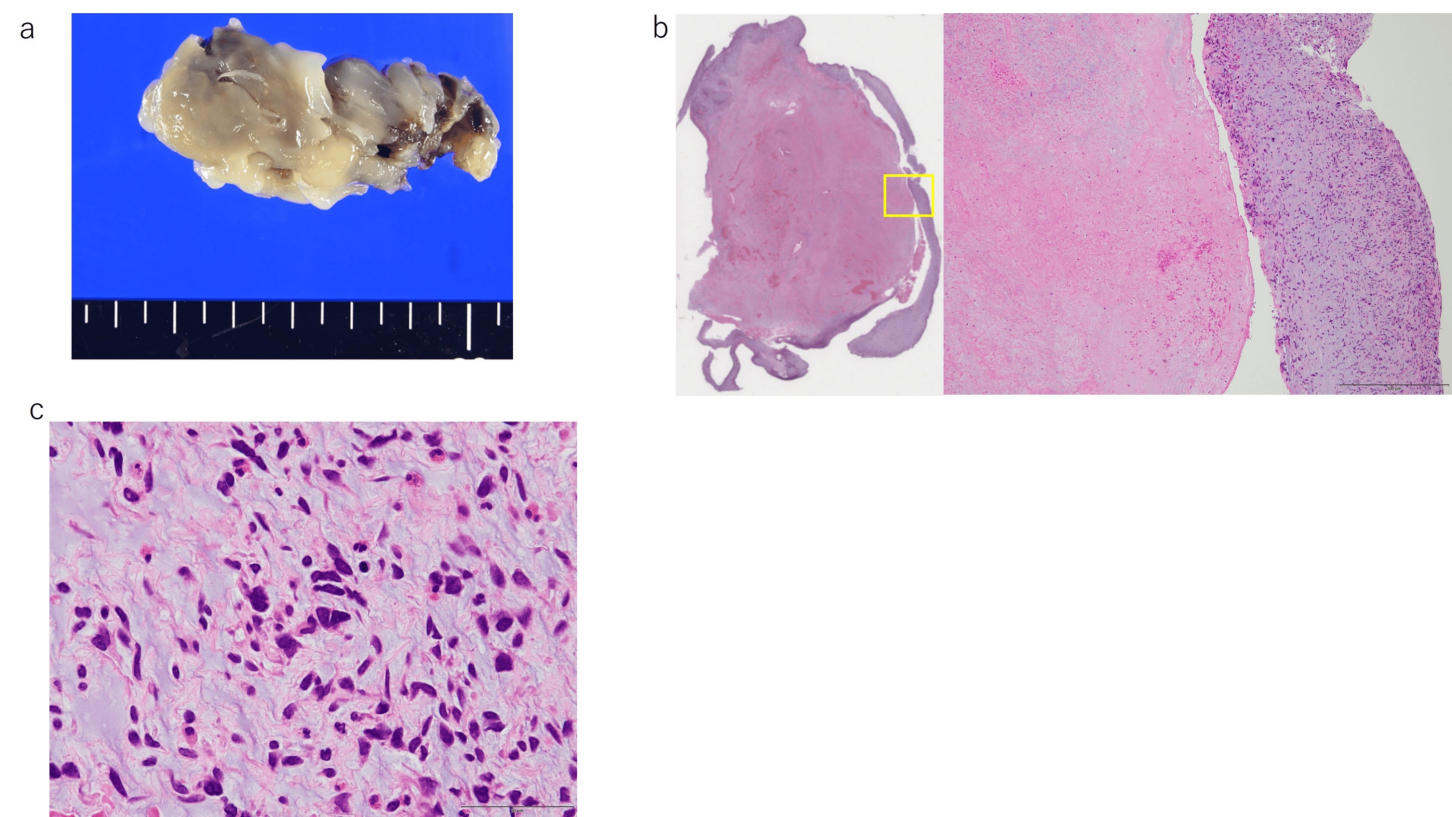
Histopathological findings of the left atrium nodule (a) Macroscopic appearance of the left atrial nodule. (b) Left: loupe; right: 4x view of the boxed area in the loupe image (H&E). Necrotic area in the center without nuclei; variable tumor cells only at the periphery. (c) Area of variable tumor cells (H&E, 40x)

**Figure 5:**
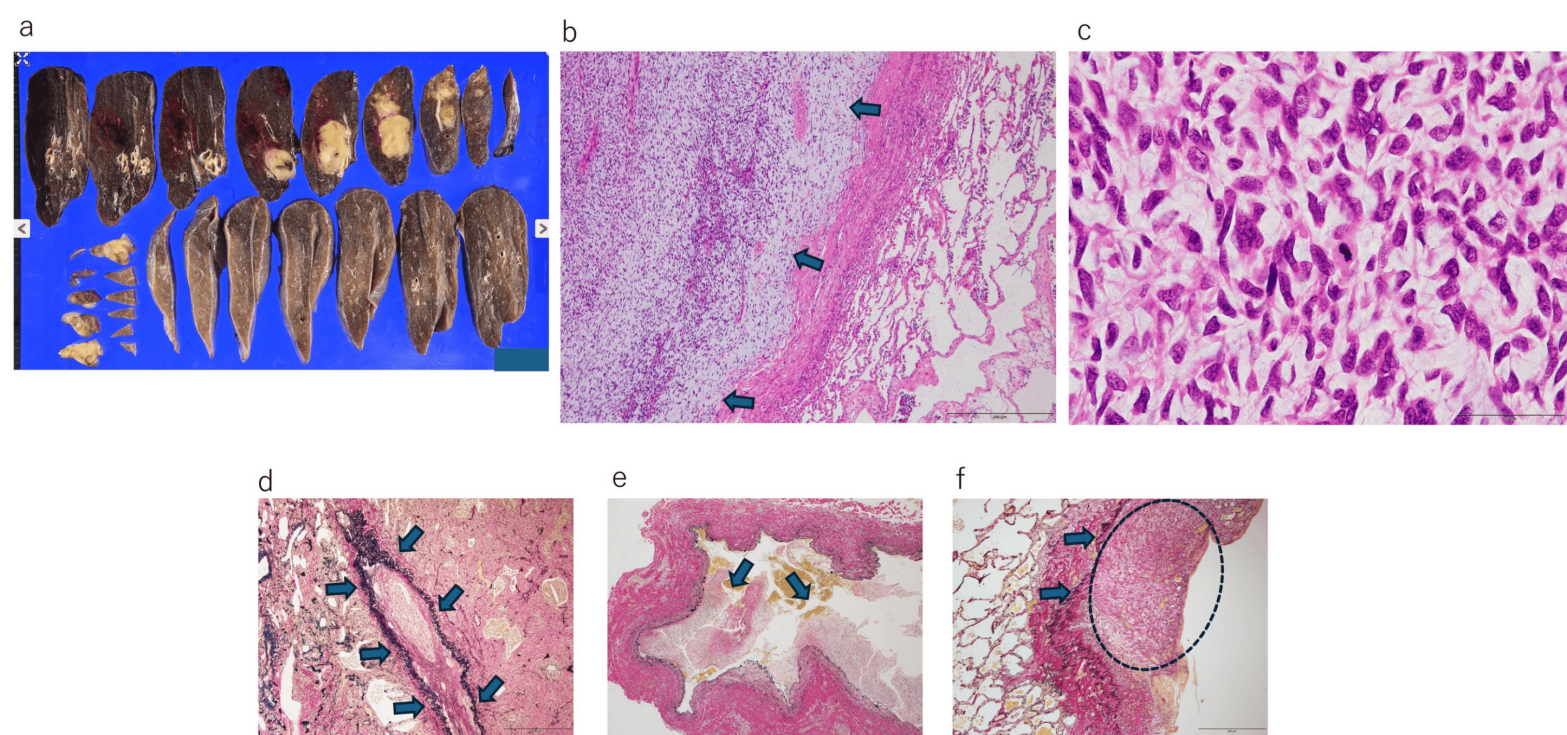
Histopathological findings of the right lower lobe of the lung (a) Macroscopic appearance of the right lower lobe of the lung. (b) Lung metastasis (H&E, 4x). Inside arrows: tumor tissue; outside arrows: normal lung tissue. (c) Lung metastasis (H&E, 40x). It is consistent with a malignant phyllodes tumor. (d) The area inside the arrows indicates a vein with intraluminal tumor invasion (EVG, 4x). (e) Tumor is also observed within the lumen of the pulmonary vein (EVG, 4x). (f) Tumor spread outside the pleura (arrows) is seen in the circled area (EVG, 4x) EVG: Elastica van Gieson

Gross examination of the left atrial mass revealed a sticky, white tumor with central necrosis (Figures 4a, 4b). Histologically, the lesion demonstrated atypical spindle cell proliferation within a myxoid stroma, consistent with metastatic malignant phyllodes tumor (Figure 4c). The right lower lobe specimen contained two tumors measuring 5.0 × 2.5 cm and 2.9 × 2.9 × 2.1 cm (Figure 5a), which showed histologic features identical to those of the atrial lesion (Figures 5b, 5c). Venous invasion (Figure 5d), extension toward the pulmonary hilum (Figure 5e), and extrapleural spread (Figure 5f) were also observed. A 2.8 × 1.1 × 1.1 cm lesion in the left upper lobe partial resection specimen demonstrated similar morphology, confirming metastatic malignant phyllodes tumor.

The postoperative course was uneventful, and the patient was discharged on postoperative day 10. She subsequently began single-agent doxorubicin therapy; however, the disease progressed after five cycles. She was later hospitalized with dyspnea and anorexia, and active treatment was discontinued. She passed away six months after surgery.

## Discussion

Phyllodes tumors, as in this case, may initially present as benign but recur locally and undergo malignant transformation [3]. Recurrence and multifocality are associated with increasing malignancy. This case demonstrated disease progression over a relatively short period, with local recurrence occurring two years after initial surgery and distant metastases developing within two years after mastectomy. The relatively short interval between initial surgery, recurrence, malignant transformation, and the development of distant metastases suggests an aggressive biological behavior in this case. Risk factors for recurrence include positive surgical margins, large tumor size, histological subtype, patient age, and necrosis. In particular, positive margins are associated with a fourfold increase in recurrence risk in multivariate analysis of 172 cases [4]. Tumorectomy without adequate margins is linked to higher local recurrence [5]. Therefore, breast-conserving surgery with ≥1 cm margins or mastectomy is recommended. In this case, the initial tumorectomy likely led to recurrence and malignant transformation. The initial CNB performed at the previous institution before the first surgery had already resulted in a diagnosis of a phyllodes tumor. Even if the tumor was classified as benign, it is conceivable that selecting a surgical approach that ensured adequate resection margins could have reduced the risk of local recurrence. Therefore, when performing surgery for phyllodes tumors, careful selection of the surgical procedure with consideration of recurrence risk is crucial.

The rate of distant metastasis in malignant phyllodes tumors ranges from 4.7% to 23% [6]. Prognosis in metastatic cases is poor, with a five-year survival rate of 47.8% reported in Japan [7]. Metastasis typically occurs via the hematogenous route, most commonly affecting the lungs, bones, and liver [2]. No standard therapy exists for metastatic disease. According to breast cancer treatment guidelines, systemic therapy is generally based on soft tissue sarcoma protocols, with doxorubicin commonly used as first-line treatment, though supporting evidence is limited [8]. Some cases have reported success with radiotherapy [9] or repeated surgical resection of metastases, leading to long-term survival [10].

A literature search via Ichushi-Web and PubMed revealed only 12 reported cases (excluding conference abstracts) of intracardiac metastasis of malignant phyllodes tumor worldwide [6,9,11-20]. Of the 12 cases, four lacked outcome data; in all others, patients died within one year of diagnosis of intracardiac metastasis (Table 1).

**Table 1:** Reported cases of intracardiac metastasis POD: postoperative day

Author	Year	Age	Time from initial surgery to intracardiac metastasis	Tumor location	Treatment	Outcome
Fleisher et al. [11]	1990	46	2 years 6 months	Left atrium	Surgery	Died 11 months after surgery
Kazuhiro et al. [12]	1998	47	8 months	Right ventricle	Surgery	Died 15 days after surgery
Jackson et al. [13]	2009	69	Unknown	Left atrium	Surgery	Survived 1 year post-op; thereafter unknown
Nakatsu et al. [14]	2010	65	9 years	Right ventricle	Surgery	Died on POD77
Garg et al. [15]	2011	35	3 years	Right ventricle	Surgery	Died on POD8
Bhambhani et al. [16]	2014	50	1 year	Left atrium	Surgery	No perioperative death; follow-up unknown
Goh et al. [17]	2014	Unknown	11 months	Right ventricle	Surgery	Died 8 months after surgery
Yoshidaya et al. [18]	2015	38	4 months	Right ventricle	Surgery	Died on POD66
Tomohiro et al. [9]	2018	56	25 years	Right atrium	Radiotherapy	Died 10 months after diagnosis of metastasis
Chen et al. [19]	2020	60	4 years 1 month	Right ventricle	Surgery	Survived 1 year post-op; thereafter unknown
Ikram et al. [20]	2022	37	10 years	Left atrium	Surgery	Died 3 months after surgery
Momono et al. [6]	2022	47	1 year 4 months	Right atrium	Surgery	Unknown
Present case			7 years	Left atrium	Surgery	Died 6 months after surgery

In the present case, the left atrial metastasis was incidentally detected on follow-up CT; however, previous reports have described cases in which intracardiac metastasis was identified following the onset of symptoms such as dyspnea or chest pain [12-16,18,20]. When thoracic symptoms develop in patients with malignant phyllodes tumors, the clinical course may be rapidly fatal. Although rare, intracardiac metastasis should therefore be considered in the differential diagnosis. To prevent critical conditions caused by cardiac dysfunction or embolic events, surgical resection of intracardiac metastatic lesions is required. In this case, resection of the metastatic lesions allowed us to avoid such potentially lethal complications and reduced the risk that cardiac dysfunction would preclude the administration of systemic therapy. As a result, postoperative doxorubicin therapy could be safely administered. Among the reported cases, only one case clearly documented the administration of postoperative systemic therapy following surgical resection of intracardiac metastasis, in which eribulin was used [9]. There have also been reports in which radiotherapy was effective for metastatic lesions, suggesting that it may be a useful option in patients for whom general anesthesia is difficult because of poor general condition or comorbidities. In addition, some reports have demonstrated long-term survival following resection of metastatic lesions. With the future development of more effective systemic therapies, a multidisciplinary treatment approach-including surgical intervention-may offer the potential for long-term survival in selected patients.

## Conclusions

We experienced a rare case of malignant phyllodes tumor with bilateral pulmonary and left atrial metastases treated surgically. Phyllodes tumors can recur locally and transform into malignant forms even when initially benign, emphasizing the importance of appropriate surgical planning. Although no standard therapy exists for metastatic cases, surgical resection and radiotherapy have shown benefit in select cases. The future development of effective systemic therapies may enable long-term survival through multimodal treatment strategies.
